# Perceived control, academic performance and well-being of Ghanaian college students with disability

**DOI:** 10.4102/ajod.v1i1.34

**Published:** 2012-10-16

**Authors:** Frances E. Owusu-Ansah, Peter Agyei-Baffour, Anthony Edusei

**Affiliations:** 1 Department of Behavioural Sciences, Kwame Nkrumah University of Science and Technology, Ghana; 2 Department of Community Health, Kwame Nkrumah University of Science and Technology, Ghana

## Abstract

**Background:**

Empirical evidence abounds showing the impact of perceived control on subjective well-being in several spheres of functioning, including academic performance. At tertiary institutions, such as the Kwame Nkrumah University of Science and Technology, Ghana, little is known about the needs of students with disabilities, as very few persons with disabilities attend institutions of higher learning.

**Objectives:**

This study examined the relationship between perceptions of control and the academic and subjective well-being of students with disabilities.

**Method:**

A total of 69 students with disabilities participated in this cross-sectional descriptive study. Using trusted control and subjective well-being scales, data were subject to descriptive analyses.

**Results:**

Consistent with previous works, perceived control increased with increased subjective well-being, moderated by gender. In addition, forms of secondary control appeared to aid primary control in the tenacious pursuit of goals. However, neither perceived control nor self-esteem was predictive of academic performance.

**Conclusion:**

Limitations of sample size notwithstanding, the findings of the study can be considered provocative. Implications for clinical utility in facilitating context-specific interventions for this marginalised group are discussed. Replication with a larger sample size in other tertiary institutions is suggested for future work.

## Introduction

Empirical evidence that consistently shows that an individual’s sense of perceived control impacts on his or her psychological and physical well-being abounds. A greater sense of control over events affecting one’s life has been associated with positive effects in several spheres of life spanning academic achievement, attenuation of stress, self-concept, better coping skills and subjective well-being (George 2010; Owusu-Ansah 2009; Ryan *et al*. 2003). Likewise, a fatalistic perception that one’s life is controlled by external forces has been associated with negative outcomes (Langdon & Talbot 2006).

Subjective well-being is about being happy and satisfied with the way one lives one’s life. But what really makes people happy and/or satisfied? Evidence suggests that in the pursuit of happiness people make choices that have maximising utility (Kiefer 2008); choices that they believe will add value to their lives or make them happy although these may not necessarily reflect their ‘true’ preferences (Kahneman & Krueger 2006). In other words, people sometimes do what they *have* to do in order to do what they *want* to do. A personal sense of control is necessary to negotiate these, sometimes conflicting, motivations for choices.

Control can be perceived and exercised as primary or secondary. Primary control aims to change the environment so that it fits with the individual’s needs and desires. It is assimilative and outwardly directed as the individual takes ‘whatever means’ necessary, direct or indirect, to persist in the pursuit of the desired goals. There are two forms of primary control: selective and compensatory. Tenacious engagement of the environment through one’s own efforts is selective, whilst the use of secondary resources to change the environment to attain one’s goals is compensatory.

Secondary control, unlike primary, is accommodative and involves attempts to fit in with the environment. It does not directly seek to alter the environment. Instead, it focuses on cognitive or motivational changes within the self. Positive reappraisal is a form of secondary control as it attempts to make sense of or find some positive meaning in an otherwise difficult or negative situation. Social comparison is another form of secondary control, whereby one compares the self with same or similar others (for example, persons of the same age, gender or condition) to enhance a perceived sense of control. Likewise, control is sometimes attained by disengaging from prior goals or adjusting and rescaling values attached to unattainable goals. This form of secondary control refers to lowering aspirations. Sometimes this is the best thing one can do to promote well-being and maintain some semblance of sanity in seemingly impossible situations (Wrosch & Lachman 2000).

Several studies have examined the effects of perceived control in the lives of persons with disabilities (PWDs). Many of these have shown that PWDs, mostly children, exhibit greater externalised perceptions of control than children without disabilities, with negative effects on their academic performance (Chapman & Boerman 1979; Firth, Cunningham & Skues 2007; Roger & Saklofske 1985) Moreover, some findings suggest that perceived control is moderated by both disability and age. Older persons with disabilities perceive themselves to have less control, especially primary control, than younger persons (Kempen *et al*. 2005).

Studies that have examined the relation of perceived control to subjective well-being of PWDs consistently show positive effects in PWDs who perceive themselves as able to control meaningful elements in their environment (Dunn 1996; Furhrer 1994; Kloomok & Cosden 1994). Although some studies have failed to confirm previous findings (Savell 1991), others clearly suggest that how a person thinks about the self, relative to the environment, has important behavioural consequences and that there is greater subjective well-being in the lives of PWDs who perceive themselves able to control meaningful elements in their environment (Fisher & Johnstion 1996).

### Justification of the study

Many studies have looked at perceived control, and/or subjective well-being of PWDs in relation to academic performance. However, most of these have focused on children (Chapman & Boerman 1979; Firth *et al*. 2007; Roger & Saklofske 1985). The relationship of perceived control and subjective well-being to the academic performance of PWDs who attend higher institutions of learning is less researched. More importantly, very little is known about the lives of tertiary students with disabilities within sub-Saharan Africa. Yet evidence abounds to suggest that a perception of self as agentic (that is, a perception of self as capable to effect a desired outcome) has multiple positive effects (Dunn 1996; Kloomok & Cosden 1994).

Therefore, the present study examined the relationship between perceived control and subjective well-being and the academic performance of PWDs at the Kwame Nkrumah University of Science and Technology (KNUST) in Kumasi, Ghana. Information from this study could inform the design of context-specific programmes aimed at enhancing the general well-being and academic performance of PWDs at tertiary institutions such as KNUST. This is an aim consistent with the goals of the Centre for Disability and Rehabilitation Studies (CEDRES) housed on the KNUST campus and the first of its kind in the sub-region. Since perceived control is important to advancement in all spheres of life, including subjective well-being, PWDs who see themselves as agentic, and thus feel empowered, are more likely to be active participants of changes needed to improve their circumstances on the university campus. However, how they perceive themselves with reference to controlling meaningful life events must first be understood. More importantly, understanding PWDs’ self-perceptions would facilitate a best-fit, context-specific and effective intervention for their academic, social and clinical needs. The study sought to answer the following specific questions:

- Do students with disabilities hold primary or secondary forms of perceived control?
- Are the specific types of perceived control differentially associated with academic performance and subjective well-being amongst students with disabilities?
- Do students with greater general perceived control have better academic grades and/or do they enjoy greater subjective well-being?

The objective of the study was to determine the relationship between perceived control and academic performance and the subjective wellbeing of PWDs at KNUST for the development of effective, context-specific interventions. Consequently, the specific objectives were to:

- determine the relationship between general perceived control and academic performance
- determine the relationship between general perceived control and subjective well-being
- inform the choice of clinical strategies and interventions for effective counselling
- inform recommendations for advising students with disabilities at a tertiary institution such as KNUST.

Based on existing literature, a positive relationship between perceptions of control, academic performance and subjective well-being was expected. Although no prediction about primary and secondary control perceptions was made, it is likely that, given environmental constraints, there would be more secondary control perceptions relative to primary control amongst students.

### Significance of the study

The significance of this study lies in the data that it provides about students with disabilities at KNUST. Insights with regard to their self-perceptions can inform appropriate and context-specific assistive response from the university.

## Method

### Setting

KNUST is one of the leading tertiary institutions in Ghana and the largest in the Kumasi metropolis. It has close to 30 000 students attending varied graduate and undergraduate programmes. The university also houses CEDRES and a hospital that serves students, staff and their dependents, as well as the general public. KNUST is yet to become fully disability friendly as far as accessibility to the built environment, organised services for students with disabilities and data on students with disabilities are concerned.

### Sample

The study focused on students with disabilities at the KNUST campus in Kumasi, Ghana. Participants were approached through their respective student representative bodies, such as the Students’ Representative Council, the KNUST branch of the Ghana Society for Physically Disabled, and the office of the Dean of Students. With assistance from the Office of the Dean of Students, a campus-wide count of students with disabilities was conducted in which students were asked to indicate whether they had any type of disability and, if applicable, to provide their contact information. Students who responded were later contacted and their voluntary participation in the study was requested. Although many students were self-identified as PWDs, only those with visible impairments of high to moderate severity (such as mobility and visual impairment) appeared to respond favourably in the follow-up solicitation for participation in the study.

### Instruments and procedure

Psychological instruments used for this cross-sectional descriptive study consisted of two scales that assessed perceived control and two subjective well-being scales. The former included the Heckhausen control scales (Heckhausen & Schulz 1995) and Rotter’s Internal-External control scale (Lefcourt 1983; Rotter 1966;), whilst the latter involved Diener’s Satisfaction with Life scale (Diener 2000; Diener *et al*. 1985) and a Psychological Well-being scale (Owusu-Ansah 2008). Rosenberg’s Self-esteem scale (Crandal 1973; Rosenberg 1965) was added to assess participants’ level of self-esteem. The Heckhausen scale consists of 30 items rated on a seven-point scale and reflects the extent to which participants perceive themselves to be agentic as far as controlling meaningful things in their environment. Rotter’s scale is a 23-item scale, excluding fillers. Items reflecting internal locus of control receive a score of 0 (zero), whilst items that reflect an external locus of control are scored 1. Scores between 0 and 11 reflect endorsement of items with an internal locus of control and scores between12 and 23 reflect endorsement of items with an external locus of control. Higher scores therefore indicate a more externalised locus of control.

Diener’s scale includes five items rated on a seven-point scale, with higher scores indicating greater satisfaction with one’s life and an index of subjective well-being. The Psychological Well-being scale consists of 25 items, also rated on a seven-point scale, which provide a general appraisal of participants’ perception of well-being in several spheres of life. The Rosenberg scale has nine items rated on a four-point scale, with higher scores reflecting a higher level of self-esteem. Demographic data and students’ grade-point average or cumulative weighted average (CWA), which was used as the index for academic performance, were also obtained.

All scales, structured as paper–pencil questionnaires, were put together in a single packet for administration to the participants after they had read, understood and signed a consent form, and all questions and/or concerns about the study were answered. Participation was voluntary without any coercion. To ensure quality of the data collection procedure, research assistants were trained for consistency in the administration of questionnaires. Visually impaired students were assisted in the completion of questionnaires by having research assistants read the questions to them and mark their choices.

## Results

A total of 69 students with disabilities, from graduate and undergraduate study programmes, participated in the study. Ages ranged from 19 to 54 years (mean = 25.4, SD = 8.6), with more male than female participants. Physical disabilities of mobility predominated, followed by visual, auditory and speech disabilities, in this order. Study programmes included English, Physics, Agricultural Studies, Nursing, Actuarial Science, Industrial Art and Human Biology. The CWA, which was used as an index of academic performance, ranged from 45.1 to 70.4 (mean = 49.6 and 55.2 for male and female students, respectively), with no significant difference between male and female participants (*p* < 0.41). The demographic characteristics of participants are presented in Table 1.

**TABLE 1:** Demographic characteristics of participants (*N* = 69).

Characteristics	Sample size (*n*)	Percentage (%)
**Age^a^ (years)**		
19–29	56	82.6
30–39	6	8.5
40–49	3	4.2
50–59	3	4.2
**Gender**		
Male	52	75.4
Female	17	24.6
**Disability type^b^**		
Mobility	25	36.2
Visual	13	18.8
Auditory	11	14.6
Speech	8	11.6
**Year of study**		
First year	15	21.7
Second year	43	62.3
Third year	10	14.5
Fourth/Graduate	9	13.0
**CWA^c^**		
40–49	3	4.2
50–59	19	26.6
60–69	28	39.2
70–79	2	2.7

CWA, cumulative weighted average.

a One participant did not indicate age.

b A total of 12 participants (17.4%) did not indicate their type of disability.

c A total of 17 participants (24.4%) did not indicate their cumulative weighted average.

All measures evidenced desirable coefficient alphas, reflective of their internal consistency reliability (see Table 2). Measures of subjective well-being evidenced good construct validity, reflected in a significant positive correlation (*r* = 0.33, *p* < 0.001). The following findings were obtained with regard to the specific study questions:

- Perceived control and well-being measures showed positive associations, as predicted, although not significant (*r* = 0.12 and *r* = 0.13, respectively). In other words, indications of well-being increased with increases in perceptions of control.
- Regressions of control measures and well-being measures, including self-esteem, on academic performance were not significantly predictive. Students were not significantly differentiated by their level of perceived control, well-being or self-esteem as far as academic performance was concerned.

**TABLE 2:** Descriptive statistics and internal consistency reliabilities for control and well-being measures.

Measures	Mean	SD	Median	Minimum	Maximum	Possible range	Range	Coefficient alpha
PC	4.9	0.6	4.7	3.2	5.8	1–7	2.6	0.7
RLCS	9.4	4.2	9.0	0	22.0	0–1	22.0	0.9
SWLS	4.1	1.3	4.2	0.8	7.0	1–7	6.2	0.7
PWB	3.9	0.6	3.8	2.9	5.0	1–7	2.0	0.8
RSE	2.7	0.3	2.8	1.4	3.6	1–4	2.1	0.8

PC, Perception of control; PWB, Psychological Well-being; RLCS, Rotter’s Locus of Control; RSE, Rosenberg Self-esteem Scale; SD, standard deviation; SWLS, Satisfaction With Life Scale.

Although there was no significant difference in the perceptions of primary and secondary forms of control amongst students, gender made a difference. A *t*-test of participants’ average scores revealed that male and female students differed significantly in the perception of secondary control, specifically in the perception of lowering aspiration (*p* < 0.002) and social comparison (*p* < 0.03). Female students significantly tended to engage in both types of control, more so than their male counterparts. That is, female students were more likely than male students to lower their aspirations and compare themselves to others.

Additional information from examination of the inter-construct relationships (that is, an examination of how the types of control used measured same or different aspects of control) showed interesting findings. A negative relationship between primary control and lowering aspiration not only showed a difference in the constructs but also suggested that those who engaged in tenacious pursuit of goals through perceptions of primary control were less likely to lower their life aspirations (*r* = –0.22, *p* < 0.05). Instead, primary control perceptions were positively associated with social comparison (*r* = 0.22, *p* < 0.05) and positive reappraisal (*r* = 0.57, *p* < 0.01). Finally, those who lowered aspirations to meet goals significantly tended to compare themselves with others (*r* = 0.23, *p* < 0.05). In other words, persons who believed in their ability to change their environment through their own efforts (primary control) were more likely to compare themselves with others like them (social comparison) and to find the ‘silver lining’ in even negative situations (positive reappraisal).

Further analysis of variance to explore the relationship between perceived control (that is, the Rotter’s Locus of Control measure), self-esteem and disability type revealed no significant difference between type of disability, self-esteem (*p* < 0.12) and perception of control (*p* < 0.37). In other words, the type of disability students experienced was not significantly related to either their self-esteem or their perceived control. On Rotter’s Locus of Control measure, all students were internally oriented and did not differ significantly from each other (see Table 3). It appears that students with different disabilities were generally similar in the way they viewed themselves and how much control they believed they had in controlling meaningful happenings in their lives. Therefore, whereas gender made a difference in the perception of some types of control (secondary control on the Heckhausen control measures), type of disability did not.

**TABLE 3:** Summary of internal and external locus of control scores by type of disability.

Type of disability	Locus of control score	SD
Mobility	10.3	5.1
Visual	9.2	3.4
Auditory	11.3	2.8
Speech	7.9	3.6

SD, standard deviation.

It was noteworthy that many participants denied membership of the local association for students with disabilities (59.4% vs. 21.7% who acknowledged membership), whilst others did not answer this question at all (18.8%, 13). Although the majority received a bursary for students with disabilities (62.3% vs. 21.7%), some did not answer this question (15.9%, 11).

## Ethical clearance

Ethical approval for the study was obtained from the Committee for Human Research, Publications and Ethics at KNUST.

## Discussion

In this study increases in perceived control were associated with increases in life satisfaction and well-being. Put differently, students who perceived themselves to have greater control over meaningful happenings in their lives also reported greater levels of life satisfaction and well-being. This finding is consistent with previous studies that showed positive relationships between perceived control and well-being (Dunn 1996; Fisher & Johnstion 1996; George 2010; Grob *et al*. 1996; Kloomok & Cosden 1994; Owusu-Ansah 2008; 2009; Pallant 2012; Ryan *et al*. 2003). Also consistent with earlier works (Kempen *et al*. 2005; Lang & Heckhausen 2001), participants of this study, who were predominantly young, believed that important happenings in their lives were internally controlled, as evidenced by scores on the Rotter scale. Understandably, PWDs who have managed to attend a tertiary institution such as KNUST despite the numerous challenges they face within the Ghanaian educational system (Danso, Owusu-Ansah & Alorwu 2012), are likely to have a sense of accomplishment and a level of life satisfaction as a result. However, there was a greater dispersion in the level of perceived control experienced by those with mobility impairments relative to the other disabled groups, as evidenced by the deviation of their scores from the mean. It may be possible that the physically challenged felt more constrained and dissatisfied because of the physical inaccessibility of the KNUST campus. Being visually aware of existing facilities and opportunities yet barred from full participation because of a lack of accessibility for the mobility impaired may be particularly frustrating.

It was surprising to find that neither perceived control nor subjective well-being was predictive of academic performance given the positive associations between perceived control and academic performance found in some studies (Diener & Suh 2000; Weist, Wong & Kreil 1998). In other words, while some earlier works have shown that greater perceived control and/or subjective well-being predicted academic performance, the findings of this study failed to confirm that. However, it is noteworthy that the literature in this area has presented mixed findings, which may be attributable to differences in study samples. As in the present study, some studies (e.g. Stupnisky *et al*. 2007) have found that self-esteem, an index of well-being, was not predictive of grade-point average in first-year college students. However, unlike the present study, their sample was first-year college students without disabilities. Also, comparative studies of controls and children with disabilities have reported negative associations with academic performance (Chapman & Boerman 1979; Dunn 1996; Firth *et al*. 2007; Kloomok & Cosden 1994; Roger & Saklofske 1985). That is, these studies did not observe positive and predictive associations between perceived control and academic performance. It is fair to say that unlike these studies, which report greater externalisation of control in children with disabilities, with adverse effects on their academic performance, participants in the present study were neither young children nor externally localised in their perceptions of control. Therefore it is possible that the type and size of the sample in the present study may have contributed to the lack of significant and predictive associations between perceived control and academic performance.

The finding of positive associations between positive reappraisal and social comparison with primary control in the present study, suggests that these forms of secondary control bolster tenacious pursuit of goals. In other words, in the persistent pursuit of life’s goals it helps to be able to see the brighter side of seemingly negative happenings and to consider one’s situation to be better than that of similar others. A positive reappraisal does not necessarily take away present challenges, but may give a new perspective and impetus to keep striving despite challenges and constraints.

Honing the skills for positive reappraisal in PWDs might have therapeutic benefits, especially when the individual desires to control meaningful personal life events. Likewise, it appears that being able to appreciate one’s circumstances by considering oneself to be in a better situation than similar others or those with a worse alternative can motivate one to continue in the pursuit of life goals. The findings of this study, although preliminary, suggest that a consideration of gender rather than type of disability might be important when counselling students with disabilities. Understanding the potential cultural dynamics that may underlie why a female student with disability would be more likely to lower her life aspirations and engage in social comparison as a means of enhancing perceived control would inform the choice of therapeutic interventions and/or counselling style.

## Conclusion

The findings of this study open interesting questions for further investigation with potential clinical utility, the limitations of the small sample size notwithstanding. Given the small sample size and possible cultural perceptions of gender and disability that may have contributed to the results, the findings can be considered provocative rather than definitive. It is therefore recommended that the study be replicated with a larger sample size in other tertiary institutions, whether in Ghana or outside Ghana, and to include a control group for more informative and effective comparative analyses. Since the observed correlations were generally in the expected direction, findings from a larger sample with a control group would be more informative and illuminating. More importantly, a better understanding of the way young adults with disabilities at tertiary institutions perceive themselves and how this relates to their academic achievement will have numerous clinical, social, and economic implications.
